# Supplementary material for: A Case-Control Analysis of the Impact of Venous Thromboembolic Disease on Quality of Life of Patients with Cancer: Quality of Life in Cancer (Qca) Study
Source: Cancers (Basel). 2019 Dec 26;12(1):75. doi: 10.3390/cancers12010075 (PMC7017186; doi:10.3390/cancers12010075)
Supplement: Supplementary file 1 [file cancers-12-00075-s001.zip › cancers-673688-supplementary.doc]

**Supplementary Table 1. Recruitment per center.**

| **CENTER** | **TYPE OF PATIENT INCLUDED** | **NUMBER OF PATIENTS** |
| --- | --- | --- |
| NISA Hospital, Sevilla, Spain. | Controls | 37 |
| Hospital Universitario Virgen de Valme, Sevilla, Spain. | Cases and controls | 61 |
| Hospital Universitario Virgen Macarena, Sevilla, Spain. | Cases | 26 |
| Hospital de Orense, Orense, Spain. | Controls | 15 |
| Hospital Clínico San Carlos, Madrid. | Controls | 3 |
| Hospital de Jerez de la Frontera, Cádiz, Spain. | Controls | 14 |
| Hospital Clínic, Barcelona, Spain | Controls | 27 |
| Hospital de Galdakao-Usansolo, Bizkaia, Spain | Cases | 6 |
| H. U. Puerto Real, Cádiz, Spain | Cases and controls | 9 |
| Hospital Universitario Central de Asturias, Oviedo, Spain | Controls | 24 |
| Hospital General Universitario Gregorio Marañón, Madrid, Spain | Controls | 80 |
| Hospital San Pedro Alcantara, Caceres, Spain | Controls | 55 |
| Hospital Virgen del Rocio, Sevilla, Spain | Cases and controls | 66 |

**Supplementary Table 2. Marital status, education status, employment status, and gross income of the study patients**

| **Variable** | **Cases**  **(cancer with VTE)** | **Controls**  **(cancer without VTE)** | **Entire cohort** |
| --- | --- | --- | --- |
| **Marital status (n=385)** |  |  |  |
| Single, n (%) | 6 (6.2%) | 43 (14.9%) | 49 (12.7%) |
| Married, n (%) | 73 (75.3%) | 219 (76%) | 292 75.8%) |
| Separated, n (%) | 3 (3.1%) | 9 (3.1%) | 12 (3.1%) |
| Divorced, n (%) | 4 (4.1%) | 10 (3.5%) | 14 (3.6%) |
| Widowed, n (%) | 11 (11.3%) | 7 (2.4%) | 18 (4.7%) |
| **Education status (n=360)** |  |  |  |
| No education, n (%) | 4 (4.2%) | 8 (3%) | 12 (3.3%) |
| Primary, n (%) | 45 (47.4%) | 77 (29.1%) | 122 (33.9%) |
| Secondary, n (%) | 9 (9.5%) | 38 (14.3%) | 47 (13.1%) |
| Vocational training, n (%) | 13 (13.7%) | 30 (11.3%) | 43 (11.9%) |
| Middle school, n (%) | 6 (6.3%) | 41 (15.5%) | 47 (13.1%) |
| University, n (%) | 14 (14.7%) | 60 (22.6%) | 74 (20.6%) |
| Other studies, n (%) | 4 (4.2%) | 11 (4.2%) | 15 (4.2%) |
| **Employment status (n=354)** |  |  |  |
| Employed, n (%) | 13 (14.1%) | 69 (26.3%) | 82 (23.2%) |
| Retired, n (%) | 57 (62%) | 144 (54.9%) | 201 (56.8%) |
| Unemployed, n (%) | 8 (8.7%) | 26 (10%) | 34 (9.6%) |
| Student, n (%) | 0 (0%) | 5 (1.9%) | 5 (1.4%) |
| Homemaker, n (%) | 14 (15.2%) | 16 (6.1%) | 30 (8.5%) |
| Other working conditions, n (%) | 0 (0%) | 2 (0.8%) | 2 (0.6%) |
| **Gross income (n=233)** |  |  |  |
| 19,999 Euros or less, n (%) | 41 (67.2%) | 98 (57%) | 139 (59.7%) |
| 20,000-39,999 Euros, n (%) | 17 (27.9%) | 55 (32%) | 72 (30.9%) |
| 40,000-59,999 Euros, n (%) | 0 (0%) | 14 (8.1%) | 14 (6%) |
| 60,000-99,999 Euros, n (%) | 3 (4.9%) | 4 (2.3%) | 7 (3%) |
| More than 100,000 Euros, n (%) | 0 (0%) | 1 (0.6%) | 1 (0.4%) |
| Abbreviation: VTE: venous thromboembolism. | | | |

**Supplementary Table 3. Differences in terms of QLQ-c30 functional and symptom scales scores between patients with pulmonary embolism and deep vein thrombosis**

| QLQ-c30 Functional scales | Pulmonary embolism | Deep vein thrombosis | Mean difference | Effect size (95% CI) | Relative efficiency (95% CI) |
| --- | --- | --- | --- | --- | --- |
| Global health status | 46.5 | 48.9 | -2.4 | 1.52 (0.8; 2.6) | -0.09 (-0.4; 0.3) |
| Physical functioning | 62.5 | 66.0 | -4.5 | 1.39 (0.8; 2.4) | -0.13 (-0.5; 0.2) |
| Role functioning | 50.3 | 56.8 | -6.5 | 1.20 (0.7; 2.0) | -0.19 (-0.6; 0.2) |
| Emotional functioning | 65.8 | 68.3 | -2.5 | 1.1 (0.7; 1.9) | -0.09 (-0.4; 0.3) |
| Cognitive functioning | 79.5 | 83.6 | -4.1 | 1.09 (0.6; 1.8) | -0.15 (-0.5; 0.2) |
| Social functioning | 60.0 | 64.0 | -4.0 | 1.09 (0.6; 1.9) | -0.11 (-0.5; 0.2) |
| QLQ-c30 Symptoms scales |  |  |  |  |  |
| Fatigue | 51.4 | 45.9 | +5.5 | 1.18 (0.7; 2.0) | 0.18 (-0.2; 0.5) |
| Nausea and vomiting | 14.1 | 14.2 | -0.1 | 0.7 (0.4; 1.3) | -0.04 (-04; 0.4) |
| Pain | 35.2 | 36.7 | -1.5 | 0.9 (0.6; 1.6) | -0.04 (-0.4; 0.3) |
| Dyspnea | 32.7 | 15.9 | +16.8* | 1.6 (0.9; 2.7) | 0.57 (0.2; 0.9) |
| Insomnia | 28.8 | 31.2 | -2.4 | 0.91 (0.5; 1.5) | -0.07 (-0.4; 0.3) |
| Appetite Loss | 27.0 | 24.7 | 2.3 | 1.13 (0.7; 1.9) | 0.07 (-0.3; 0.4) |
| Constipation | 25.8 | 27.6 | -1.8 | 1.34 (0.8; 2.3) | -0.05 (-0.4; 0.3) |
| Diarrhea | 10.9 | 18.9 | -8.0 | 0.65 (0.4; 1.1) | -0.28 (-0.6; 0.1) |
| Financial difficulties | 17.6 | 29.1 | -11.5 | 0.59 (0.3; 1.0) | -0.36 (-0.7; 0.02) |
| EQ-5D-3L |  |  |  |  |  |
| Index score | 0.53 | 0.56 | -0.04 | 1.44 (0.8; 2.5) | -0.09 (-0.5; 0.3) |
| Abbreviation: CI, confidence interval.  *p<0.05; ** p<0.001 | | | | | |

**Supplementary Table 4. Sex-related differences in terms of EORTC QLQ-c30, EQ-5D-3L, PEmb-QoL and Veines-QoL/Sym questionnaire results in patients with venous thromboembolic disease**

|  | Men | | | | | Women | | | | |
| --- | --- | --- | --- | --- | --- | --- | --- | --- | --- | --- |
| EORTC QLQ-C30 Functional scales | Cases  (cancer with VTE) | Controls  (cancer without VTE) | Mean difference | ES (95% CI) | RE (95% CI) | Cases  (cancer with VTE) | Controls  (cancer without VTE) | Mean difference | ES (95% CI) | RE (95% CI) |
| Global health status | 51.4 | 57.8 | -6.7^+^ | 1.00 (0.7; 1.7) | -0.2 (-0.5; 0.07) | 44.0 | 59.3 | -15.3** | 0.78 (0.5; 1.3) | -0.6 (-0.9; -0.2) |
| Physical functioning | 66.2 | 80.1 | -13.9** | 1.32 (0.9; 2.0) | -0.6 (-0.9; -0.3) | 62.6 | 81.3 | -18.7** | 1.36 (0.9; 2.2) | -0.9 (-1.2; -0.5) |
| Role functioning | 57.6 | 75.9 | -18.3** | 1.29 (0.8; 2.0) | -0.6 (-0.9; -0.2) | 50.0 | 72.9 | -22.9** | 1.21 (0.8; 1.9) | -0.7 (-1.0; -0.4) |
| Emotional functioning | 69.0 | 76.0 | -7.0^+^ | 1.02 (0.7; 1.6) | -0.3 (-0.6; 0.01) | 65.3 | 67.3 | -2.0 | 0.89 (0.6; 1.4) | -0.07 (-0.4; 0.2) |
| Cognitive functioning | 82.7 | 86.1 | -3.4^+^ | 1.50 (0.9; 2.4) | -0.15 (-0.5; 0.1) | 80.7 | 82.2 | -1.5 | 1.25 (0.8; 2.0) | -0.06 (-0.4; 0.3) |
| Social functioning | 64.6 | 76.3 | -11.7* | 1.32 (0.9; 2.1) | -0.4 (-0.7; -0.1) | 59.8 | 71.1 | -11.3* | 1.36 (0.9; 2.2) | -0.35 (-0.7; -0.03) |
| EORTC QLQ-C30 Symptoms scales | Cases  (cancer with VTE) | Controls  (cancer without VTE) | Mean difference | ES (95% CI) | RE (95% CI) | Cases  (cancer with VTE) | Controls  (cancer without VTE) | Mean difference | ES (95% CI) | RE (95% CI) |
| Fatigue | 44.3 | 33.4 | +10.9* | 1.4 (0.9; 2.2) | 0.38 (0.07; 0.7) | 52.7 | 34.1 | +18.6** | 1.1 (0.7; 1.8) | 0.7 (0.3; 1.0) |
| Nausea and vomiting | 10.4 | 10.5 | +1.9^+^ | 0.91 (0.6; 1.4) | 0.009 (-0.2; 0.4) | 16.1 | 12.8 | +3.2 | 1.03 (0.7; 1.7) | 0.14 (-0.2; 0.5) |
| Pain | 35.3 | 22.8 | +12.5* | 1.25 (0.8; 2.0) | 0.42 (0.1; 0.7) | 36.8 | 23.5 | +13.3** | 1.92* (1.2; 3.1) | 0.47 (0.1; 0.8) |
| Dyspnea | 24.8 | 13.4 | +11.4* | 1.70* (1.1; 2.6) | 0.42 (0.12; 0.73) | 22.2 | 12.5 | +9.7* | 1.30 (0.8; 2.1) | 0.36 (0.03; 0.7) |
| Insomnia | 27.7 | 32.0 | -4.3^+^ | 0.98 (0.65; 1.5) | -0.12 (-0.4; 0.2) | 32.7 | 31.7 | +1.0 | 0.96 (0.6; 1.5) | 0.03 (-0.3; 0.35) |
| Appetite loss | 27.7 | 25.1 | +2.6^+^ | 1.10 (0.7; 1.7) | 0.08 (-0.2; 0.4) | 23.8 | 25.7 | -1.9 | 0.95 (0.6; 1.5) | -0.05 (-0.3; 0.26) |
| Constipation | 32.2 | 22.1 | +10.1* | 1.36 (0.9; 2.2) | 0.33 (0.02; 0.64) | 21.0 | 22.1 | -1.1 | 1.08 (0.7; 1.7) | -0.03 (-0.4; 0.29) |
| Diarrhea | 12.7 | 15.7 | -3.0^+^ | 0.85 (0.5; 1.4) | -0.11 (-0.4; 0.2) | 17.5 | 17.6 | -0.1 | 1.6* (1.0; 2.5) | -0.001 (-0.3; 0.3) |
| Financial difficulties | 21.0 | 17.6 | +3.4^+^ | 1.23 (0.8; 2.0) | 0.11 (-0.2; 0.4) | 25.9 | 18.1 | +7.8 | 1.5 (0.9; 2.5) | 0.26 (-0.07; 0.6) |
| EQ-5D-3L | Cases  (cancer with VTE) | Controls  (cancer without VTE) | Mean difference | ES (95% CI) | RE (95% CI) | Cases  (cancer with VTE) | Controls  (cancer without VTE) | Mean difference | ES (95% CI) | RE (95% CI) |
| Index score | 0.59 | 0.77 | -0.19** | 1.74* (1.1; 2.8) | -0.60 (-0.9; -0.3) | 0.50 | 0.76 | -0.26** | 2.6* (1.7; 4.3) | -0.85 (-1.2; -0.5) |
| VEINES-QoL/Sym | Cases  (cancer with VTE) | Controls  (cancer without VTE) | Mean difference | ES (95% CI) | RE (95% CI) | Cases  (cancer with VTE) | Controls  (cancer without VTE) | Mean difference | ES (95% CI) | RE (95% CI) |
| VEINES-QoL | 44.7 | 51.9 | -7.2** | 0.97 (0.54; 1.6) | 0.86(0.5; 1.2) | 40.2 | 51.3 | -11.1** | 0.72 (0.4; 1.3) | 1.41 (0.9; 1.9) |
| VEINES/Sym | 45.4 | 51.4 | -6.0** | 0.93 (0.52; 1.5) | 0.61 (0.2; 1.0) | 41.7 | 51.4 | -9.7** | 0.67 (0.3; 1.2) | 1.07 (0.6; 1.5) |
| PEmb-QoL | Cases  (cancer with VTE) | Controls  (cancer without VTE) | Mean difference | ES (95% CI) | RE (95% CI) | Cases  (cancer with VTE) | Controls  (cancer without VTE) | Mean difference | ES (95% CI) | RE (95% CI) |
| Quality of life | 38.6 | 25.0 | +13.6* | 0.98 (0.5; 1.9) | 0.5 (0.1; 0.9) | 49.9 | 20.1 | 29.8** | 0.98 (0.6; 2.0) | 1.44 (0.9; 1.9) |
| Frequency of complaints | 28.0 | 22.4 | +5.6^+^ | 0.76 (0.4; 1.5) | 0.2 (-0.2; 0.6) | 39.1 | 19.0 | 20.1** | 1.0 (0.6; 2.0) | 0.9 (0.4; 1.3) |
| Limitations in daily life activities | 45.6 | 31.2 | +14.4* | 1.11 (0.6; 2.2) | 0.49 (0.05; 0.9) | 58.4 | 26.6 | 31.7** | 0.99 (0.6; 2.0) | 1.17 (0.7; 1.6) |
| Work-related problems | 63.5 | 43.9 | +19.6^+^ | 1.02 (0.6; 2.1) | 0.42 (-0.01; 0.9) | 82.0 | 33.7 | 48.3** | 0.6 (0.3; 1.3) | 1.13 (0.7; 1.6) |
| Social limitations | 32.3 | 18.3 | +14.0* | 1.65 (0.9; 3.3) | 0.47 (0.04; 0.9) | 42.0 | 11.5 | 30.5** | 1.4 (0.8; 2.8) | 1.21 (0.7; 1.6) |
| Intensity of complaints | 32.0 | 16.5 | +15.5* | 1.38 (0.8; 2.8) | 0.69 (0.2; 1.1) | 35.6 | 14.0 | 21.6** | 1.4 (0.8; 2.8) | 1.09 (0.6; 1.5) |
| Emotional complaints | 30.2 | 17.3 | +12.9* | 0.9 (0.5; 1.8) | 0.6 (0.2; 1.0) | 42.3 | 16.4 | 25.9** | 1.6 (0.9; 3.4) | 1.19 (0.7; 1.6) |
| Abbreviations: VTE: venous thromboembolism; ES: effect size; RE: relative efficiency; CI, confidence interval.  *<0.05; ** p<0.001 | | | | | | | | | | |

|  | Men | | | | | Women | | | | |
| --- | --- | --- | --- | --- | --- | --- | --- | --- | --- | --- |
| EORTC QLQ-C30 Functional scales | Cases  (cancer with VTE) | Controls  (cancer without VTE) | Mean difference | ES (95% CI) | RE (95% CI) | Cases  (cancer with VTE) | Controls  (cancer without VTE) | Mean difference | ES (95% CI) | RE (95% CI) |
| Global health status | 51.4 | 57.8 | -6.7^+^ | 1.00 (0.7; 1.7) | -0.2 (-0.5; 0.07) | 44.0 | 59.3 | -15.3** | 0.78 (0.5; 1.3) | -0.6 (-0.9; -0.2) |
| Physical functioning | 66.2 | 80.1 | -13.9** | 1.32 (0.9; 2.0) | -0.6 (-0.9; -0.3) | 62.6 | 81.3 | -18.7** | 1.36 (0.9; 2.2) | -0.9 (-1.2; -0.5) |
| Role functioning | 57.6 | 75.9 | -18.3** | 1.29 (0.8; 2.0) | -0.6 (-0.9; -0.2) | 50.0 | 72.9 | -22.9** | 1.21 (0.8; 1.9) | -0.7 (-1.0; -0.4) |
| Emotional functioning | 69.0 | 76.0 | -7.0^+^ | 1.02 (0.7; 1.6) | -0.3 (-0.6; 0.01) | 65.3 | 67.3 | -2.0 | 0.89 (0.6; 1.4) | -0.07 (-0.4; 0.2) |
| Cognitive functioning | 82.7 | 86.1 | -3.4^+^ | 1.50 (0.9; 2.4) | -0.15 (-0.5; 0.1) | 80.7 | 82.2 | -1.5 | 1.25 (0.8; 2.0) | -0.06 (-0.4; 0.3) |
| Social functioning | 64.6 | 76.3 | -11.7* | 1.32 (0.9; 2.1) | -0.4 (-0.7; -0.1) | 59.8 | 71.1 | -11.3* | 1.36 (0.9; 2.2) | -0.35 (-0.7; -0.03) |
| EORTC QLQ-C30 Symptoms scales | Cases  (cancer with VTE) | Controls  (cancer without VTE) | Mean difference | ES (95% CI) | RE (95% CI) | Cases  (cancer with VTE) | Controls  (cancer without VTE) | Mean difference | ES (95% CI) | RE (95% CI) |
| Fatigue | 44.3 | 33.4 | +10.9* | 1.4 (0.9; 2.2) | 0.38 (0.07; 0.7) | 52.7 | 34.1 | +18.6** | 1.1 (0.7; 1.8) | 0.7 (0.3; 1.0) |
| Nausea and vomiting | 10.4 | 10.5 | +1.9^+^ | 0.91 (0.6; 1.4) | 0.009 (-0.2; 0.4) | 16.1 | 12.8 | +3.2 | 1.03 (0.7; 1.7) | 0.14 (-0.2; 0.5) |
| Pain | 35.3 | 22.8 | +12.5* | 1.25 (0.8; 2.0) | 0.42 (0.1; 0.7) | 36.8 | 23.5 | +13.3** | 1.92* (1.2; 3.1) | 0.47 (0.1; 0.8) |
| Dyspnea | 24.8 | 13.4 | +11.4* | 1.70* (1.1; 2.6) | 0.42 (0.12; 0.73) | 22.2 | 12.5 | +9.7* | 1.30 (0.8; 2.1) | 0.36 (0.03; 0.7) |
| Insomnia | 27.7 | 32.0 | -4.3^+^ | 0.98 (0.65; 1.5) | -0.12 (-0.4; 0.2) | 32.7 | 31.7 | +1.0 | 0.96 (0.6; 1.5) | 0.03 (-0.3; 0.35) |
| Appetite loss | 27.7 | 25.1 | +2.6^+^ | 1.10 (0.7; 1.7) | 0.08 (-0.2; 0.4) | 23.8 | 25.7 | -1.9 | 0.95 (0.6; 1.5) | -0.05 (-0.3; 0.26) |
| Constipation | 32.2 | 22.1 | +10.1* | 1.36 (0.9; 2.2) | 0.33 (0.02; 0.64) | 21.0 | 22.1 | -1.1 | 1.08 (0.7; 1.7) | -0.03 (-0.4; 0.29) |
| Diarrhea | 12.7 | 15.7 | -3.0^+^ | 0.85 (0.5; 1.4) | -0.11 (-0.4; 0.2) | 17.5 | 17.6 | -0.1 | 1.6* (1.0; 2.5) | -0.001 (-0.3; 0.3) |
| Financial difficulties | 21.0 | 17.6 | +3.4^+^ | 1.23 (0.8; 2.0) | 0.11 (-0.2; 0.4) | 25.9 | 18.1 | +7.8 | 1.5 (0.9; 2.5) | 0.26 (-0.07; 0.6) |
| EQ-5D-3L | Cases  (cancer with VTE) | Controls  (cancer without VTE) | Mean difference | ES (95% CI) | RE (95% CI) | Cases  (cancer with VTE) | Controls  (cancer without VTE) | Mean difference | ES (95% CI) | RE (95% CI) |
| Index score | 0.59 | 0.77 | -0.19** | 1.74* (1.1; 2.8) | -0.60 (-0.9; -0.3) | 0.50 | 0.76 | -0.26** | 2.6* (1.7; 4.3) | -0.85 (-1.2; -0.5) |
| VEINES-QoL/Sym | Cases  (cancer with VTE) | Controls  (cancer without VTE) | Mean difference | ES (95% CI) | RE (95% CI) | Cases  (cancer with VTE) | Controls  (cancer without VTE) | Mean difference | ES (95% CI) | RE (95% CI) |
| VEINES-QoL | 44.7 | 51.9 | -7.2** | 0.97 (0.54; 1.6) | 0.86(0.5; 1.2) | 40.2 | 51.3 | -11.1** | 0.72 (0.4; 1.3) | 1.41 (0.9; 1.9) |
| VEINES/Sym | 45.4 | 51.4 | -6.0** | 0.93 (0.52; 1.5) | 0.61 (0.2; 1.0) | 41.7 | 51.4 | -9.7** | 0.67 (0.3; 1.2) | 1.07 (0.6; 1.5) |
| PEmb-QoL | Cases  (cancer with VTE) | Controls  (cancer without VTE) | Mean difference | ES (95% CI) | RE (95% CI) | Cases  (cancer with VTE) | Controls  (cancer without VTE) | Mean difference | ES (95% CI) | RE (95% CI) |
| Quality of life | 38.6 | 25.0 | +13.6* | 0.98 (0.5; 1.9) | 0.5 (0.1; 0.9) | 49.9 | 20.1 | 29.8** | 0.98 (0.6; 2.0) | 1.44 (0.9; 1.9) |
| Frequency of complaints | 28.0 | 22.4 | +5.6^+^ | 0.76 (0.4; 1.5) | 0.2 (-0.2; 0.6) | 39.1 | 19.0 | 20.1** | 1.0 (0.6; 2.0) | 0.9 (0.4; 1.3) |
| Limitations in daily life activities | 45.6 | 31.2 | +14.4* | 1.11 (0.6; 2.2) | 0.49 (0.05; 0.9) | 58.4 | 26.6 | 31.7** | 0.99 (0.6; 2.0) | 1.17 (0.7; 1.6) |
| Work-related problems | 63.5 | 43.9 | +19.6^+^ | 1.02 (0.6; 2.1) | 0.42 (-0.01; 0.9) | 82.0 | 33.7 | 48.3** | 0.6 (0.3; 1.3) | 1.13 (0.7; 1.6) |
| Social limitations | 32.3 | 18.3 | +14.0* | 1.65 (0.9; 3.3) | 0.47 (0.04; 0.9) | 42.0 | 11.5 | 30.5** | 1.4 (0.8; 2.8) | 1.21 (0.7; 1.6) |
| Intensity of complaints | 32.0 | 16.5 | +15.5* | 1.38 (0.8; 2.8) | 0.69 (0.2; 1.1) | 35.6 | 14.0 | 21.6** | 1.4 (0.8; 2.8) | 1.09 (0.6; 1.5) |
| Emotional complaints | 30.2 | 17.3 | +12.9* | 0.9 (0.5; 1.8) | 0.6 (0.2; 1.0) | 42.3 | 16.4 | 25.9** | 1.6 (0.9; 3.4) | 1.19 (0.7; 1.6) |
| Abbreviations: VTE: venous thromboembolism; ES: effect size; RE: relative efficiency; CI, confidence interval.  *<0.05; ** p<0.001 | | | | | | | | | | |

Supplementary Table 5. Differences in the EORTC QLQ-C30 functional scales in patients with venous thromboembolic disease according to tumor site

| EORTC QLQ-C30  Functional scales | | Global health status | Physical functioning | Role functioning | Emotional functioning | Cognitive functioning | Social functioning |
| --- | --- | --- | --- | --- | --- | --- | --- |
| Gynecologic | Cases | 30.9 | 51.4 | 26.2 | 60.3 | 80.9 | 38.1 |
|  | Controls | 56.5 | 79.8 | 73.2 | 66.3 | 80.9 | 79.6 |
|  | Mean difference | -25.6* | -28.4* | -47.0** | -6.0 | 0.0 | -41.5* |
|  | Effect size (95% CI) | 0.45 (0.1; 2.3) | 0.84 (0.3; 4.3) | 0.78 (0.3; 3.9) | 0.89 (0.3; 4.5) | 0.35 (0.1; 1.8) | 1.92 (0.6; 9.8) |
|  | Relative efficiency (95% CI) | -1.15 (-2.0; -0.2) | -1.2 (-2.1; -0.3) | -1.57 (-2.5; -0.6) | -0.25 (-1.0; 0.6) | 0.0 (-0.8; 0.8) | -1.61 (-2.5; -0.7) |
| Lung | Cases | 58.3 | 74.4 | 65.2 | 72.2 | 86.8 | 58.6 |
|  | Controls | 61.6 | 82.3 | 70.8 | 70.2 | 85.8 | 71.2 |
|  | Mean difference | -3.3 | -7.9 | -5.6 | +2.0 | +1.0 | -12.6 |
|  | Effect size (95% CI) | 1.01 (0.5; 2.1) | 0.72 (0.4; 1.6) | 0.93 (0.5; 2.0) | 0.90 (0.4; 1.9) | 0.75 (0.4; 1.6) | 1.93 (0.7; 2.3) |
|  | Relative efficiency (95% CI) | -0.13 (-0.6; 0.3) | -0.39 (-0.9; 0.1) | -0.17 (-0.6; 0.3) | 0.08 (-04; 0.5) | 0.05 (-0.4; 0.5) | -0.42 (-0.9; 0.07) |
| Digestive | Cases | 53.12 | 63.9 | 56.2 | 63.2 | 75.9 | 67.9 |
|  | Controls | 56.72 | 81.3 | 76.6 | 75.3 | 87.0 | 78.6 |
|  | Mean difference | -3.6 | -17.4** | -20.4** | -12.1* | -11.1* | -10.7 |
|  | Effect size (95% CI) | 0.97 (0.5; 2.0) | 1.96* (1.0; 3.9) | 1.8 (0.9; 3.6) | 0.99 (0.5; 2.0) | 3.13* (1.7; 6.3) | 1.3 (0.7; 2.5) |
|  | Relative efficiency (95% CI) | -0.14 (-0.6; 0.3) | -0.87 (-1.3; -0.4) | -0.69 (-1.2; -0.2) | -0.4 (-0.9; -0.006) | -0.49 (-0.9; -0.03) | -0.39 (-0.8;0.07) |
| Urological | Cases | 43.3 | 68.0 | 46.6 | 61.6 | 83.3 | 70.0 |
|  | Controls | 65.1 | 86.6 | 83.3 | 77.3 | 84.7 | 77.3 |
|  | Mean difference | -21.8 | -18.6 | -36.7** | -15.7 | -1.4 | -7.3 |
|  | Effect size (95% CI) | 0.14 (0.03; 1.3) | 2.9 (0,6; 25.8) | 1.04 (0.2; 09,1) | 1.53 (0.3; 13.4) | 1.44 (0.3; 12.6) | 1.60 (0.4; 14.2) |
|  | Relative efficiency (95% CI) | -0.88 (-2.0; 0.3) | -0.89 (-2.0; 0.3) | -1.71 (-3.0; -0.4) | -0.5 (-1.6; 0.6) | -0.05 (-1.2; 1.8) | -0.20 (-1.3; 0.9) |
| Lymphomas | Cases | 50.5 | 64.7 | 52.9 | 63.0 | 80.2 | 62.5 |
|  | Controls | 55.4 | 77.3 | 75.4 | 69.8 | 78.1 | 68.9 |
|  | Mean difference | -4.9 | -12.6 | -22.5* | -6.8 | +2.1 | -6.4 |
|  | Effect size (95% CI) | 0.70 (0.3; 1.8) | 0.95 (0.4; 2.4) | 0.87 (0.4; 2.2) | 0.77 (0.3; 2.0) | 1.10 (0.5; 2.9) | 0.84 (0.4; 2.2) |
|  | Relative efficiency (95% CI) | -0.17 (-0.7; 0.4) | -0.48 (-1.1; 0.1) | -0.63 (-1.2; -0.03) | -0.2 (-0.8; 0.3) | 0.07 (-0.5; 0.6) | -0.19 (-0.8; 0.4) |
| Other sites | Cases | 38.4 | 60.0 | 51.4 | 70.7 | 83.3 | 63.9 |
|  | Controls | 59.1 | 79.8 | 74.2 | 74.3 | 86.3 | 71.9 |
|  | Mean difference | -20.7** | -19.8** | -22.8** | -3.6 | -3.0 | -8.0 |
|  | Effect size (95% CI) | 0.95 (0.5; 1.8) | 1.34 (0.7; 2.5) | 1.30 (0.7; 2.4) | 1.06 (0.6; 2.0) | 1.78* (1.0; 3.3) | 1.55 (0.8; 2.9) |
|  | Relative efficiency (95% CI) | -0.8 (-1.2; -0.3) | -0.8 (-1.2; -0.3) | -0.67 (-1.1; -0.2) | -0.15 (-0.6; 0.3) | -0.14 (-0.5; 0.2) | -0.24 (-0.7; 0.2) |
| Cases were patients with cancer and VTE, whereas controls were patients with cancer without VTE. Abbreviation: CI: confidence interval  *p<0.05; ** p<0.001 | | | | | | | |

**Supplementary Table 6. Differences in the EORTC QLQ-C30 symptoms scales in patients with venous thromboembolic disease according to tumor site**

| **EORTC QLQ-C30 Symptoms scales** | | **Fatigue** | **Nausea and vomiting** | **Pain** | **Dyspnea** | **Insomnia** | **Appetite Loss** | **Constipation** | **Diarrhea** | **Financial difficulties** |
| --- | --- | --- | --- | --- | --- | --- | --- | --- | --- | --- |
| Gynecologic | Cases | 68.2 | 21.4 | 40.4 | 28.5 | 5.5 | 42.8 | 22.2 | 47.6 | 47.6 |
|  | Controls | 38.1 | 13.2 | 30.4 | 14.8 | 40.4 | 26.4 | 28.5 | 19.8 | 23.4 |
|  | Mean difference | +30.1* | +8.2 | +10.0 | +13.7 | -34.9* | +16.4 | -6.3 | +28.8* | +24.2* |
|  | Effect size (95% CI) | 1.14 (0.4; 5.8) | 0.57 (0.2; 2.9) | 0.51 (0.2; 2.6) | 0.74 (0.2; 3.7) | 0.17 (0.05; 1.0) | 0.73 (0.2; 3.7) | 1.03 (0.3; 6.4) | 1.8 (0.6; 9.2) | 1.58 (0.5; 8.0) |
|  | Relative efficiency (95% CI) | 1.12 (0.2; 2.0) | 0.34 (-0.5; 1.2) | 0.39 (-0.4; 1.2) | 0.53 (-0.3; 1.4) | -1.12 (-2.0; -0.2) | 0.45 (-0.4; 1.3) | -0.23 (-1.1; 0.7) | 0.9 (0.02; 1.8) | 0.88 (-0.003; 1.8) |
| Lung | Cases | 36.6 | 20.8 | 25.0 | 19.4 | 34.7 | 34.8 | 20.3 | 20.8 | 26.6 |
|  | Controls | 34.4 | 12.5 | 28.4 | 11.8 | 36.8 | 25.7 | 22.2 | 12.5 | 16.6 |
|  | Mean difference | +2.2 | +8.3 | -3.4 | +7.6 | -2.1 | +9.1 | -1.9 | +8.3 | +10.0 |
|  | Effect size (95% CI) | 1.04 (0.5; 2.2) | 1.52  (0.8; 3.2) | 1.21 (0.6; 2.6) | 1.34 (0.7; 2.9) | 0.82 (0.4; 1.7) | 1.6 (0.8;3.5) | 0.95 (0.5; 2.0) | 2.29* (1.2; 4.9) | 2.23 (1.1; 4.7) |
|  | Relative efficiency (95% CI) | 0.08 (-0.4; 0.6) | 0.35 (-0.1; 0.8) | -0.12 (-0.6; 0.4) | 0.32 (-0.2; 0.8) | -0.06 (-0.5; 0.4) | 0.27 (-0.2; 0.8 | -0.68 (-0.6; 0.4) | 0.32 (0.2; 0.8) | 0.32 (-0.2; 0.8) |
| Digestive | Cases | 47.5 | 11.5 | 33.9 | 25.9 | 27.2 | 19.7 | 23.1 | 19.7 | 20.8 |
|  | Controls | 32.7 | 9.7 | 22.8 | 9.7 | 30.2 | 23.6 | 18.5 | 16.1 | 19.0 |
|  | Mean difference | +14.8* | +1.8 | +11.1 | +16.2** | -3.0 | -3.9 | +4.6 | +3.6 | +1.8 |
|  | Effect size (95% CI) | 1.69 (0.9; 3.4) | 0.98 (0.5; 2.0) | 1.34 (0.7; 2.7) | 2.6* (1.4; 5.2) | 0.99 (0.5; 2.0) | 0.58 (0.3; 1.1) | 0.74 (0.4; 1.5) | 1.8 (0.9; 3.7) | 1.00 (0.5; 2.1) |
|  | Relative efficiency (95% CI) | 0.56 (0.1: 1.0) | 0.08 (-0.4; 0.5) | 0.4 (-0.04; 0.8) | 0.66 (0.2; 1.1) | -0.09 (-0.5; 0.4) | -0.12 (-0.6; 0.3) | 0.15 (-0.3; 0.6) | 0.13 (-0.3; 0.6) | 0.06 (-0.4; 0.5) |
| Urological | Cases | 46.6 | 10.0 | 46.6 | 20.0 | 26.6 | 20.0 | 33.3 | 0.0 | 0.0 |
|  | Controls | 29.6 | 13.9 | 16.6 | 16.0 | 11.1 | 25.0 | 30.5 | 22.2 | 26.6 |
|  | Mean difference | +17.0 | -3.9 | +30.0* | +3.0 | +15.5 | -5.0 | +2.8 | -22.2 | -26.6 |
|  | Effect size (95% CI) | 1.28 (0.3; 11.3) | 0.43 (0.1; 3.7) | 3.2 (0.7; 28.4) | 2.8 (0.6; 24.8) | 0.89 (0.2; 78) | 0.72 (0.2; 6.3) | 1.70 (0.4; 14.9) | 0** (0; 0) | 0** (0; 0) |
|  | Relative efficiency (95% CI) | 0.5 (-0.6; 1.7) | -0.3 (-1.4; 0.8) | 1.2 (-0.3; 2.3) | 0.102 (-1.0; 1.2) | 0.5 (-0.6; 1.7) | -0.14 (-1.2; 1.0) | 0.07 (-1.1; 1.2) | 0.67 (-1.8; 0.5) | -0.93 (-2.2; 0.3) |
| Lymphomas | Cases | 46.1 | 11.7 | 47.0 | 21.5 | 35.3 | 19.6 | 35.4 | 10.4 | 27.1 |
|  | Controls | 34.2 | 13.2 | 16.6 | 18.8 | 28.2 | 32.4 | 29.5 | 14.0 | 15.8 |
|  | Mean difference | +11.9 | -1.5 | +30.4** | +2.7 | +7.1 | -12.8 | +5.9 | -3.6 | +11.3 |
|  | Effect size (95% CI) | 0.77 (-0.2; 0.9) | 0.64 (0.3; 1.6) | 1.57 (0.72; 3.9) | 0.74 (0.3; 1.9) | 1.15 (0.5; 2.9) | 0.51 (0.2; 1.3) | 1.38 (0.6; 3.6) | 1.34 (0.6; 3.5) | 1.2 (0.5; 3.1) |
|  | Relative efficiency (95% CI) | 0.38 (-0.2; 1.0) | -0.06  (-0.6; 0.5) | 0.91 (0.3; 1.5) | 0.09 (-0.4; 0.7) | 0.2 (-0.4; 0.8) | -0.37 (-1.0; 0.2) | 0.16 (-0.4; 0.7) | -0.15 (-0.7; 0.4) | 0.39 (-0.2; 0.9) |
| Other sites | Cases | 54.6 | 12.0 | 37.5 | 25.0 | 31.5 | 25.0 | 29.5 | 4.3 | 19.3 |
|  | Controls | 32.6 | 10.2 | 20.9 | 12.4 | 32.2 | 22.0 | 16.9 | 19.1 | 14.6 |
|  | Mean difference | +22.0** | +1.8 | +16.6** | +12.6* | -0.7 | +3.0 | 12.6* | -14.8* | +4.7 |
|  | Effect size (95% CI) | 1.34 (0.7; 2.5) | 0.99 (0.6; 1.8) | 1.85* (1.0; 3.4) | 1.66 (0.9; 3.0) | 1.16 (0.6; 2.1) | 1.77 * (1.0; 3.3) | 2.23* (1.2; 4.2) | 0.22 (0.1; 0.4) | 1.22 (0.7; 2.4) |
|  | Relative efficiency (95% CI) | 0.77 (0.3; 1.2) | 0.09 (-0.3; 0.5) | 0.58 (0.15; 1.0) | 0.42 (0.007; 0.8) | -0.02 (-0.4; 0.4) | 0.09 (-0.3; 0.5) | 0.43 (0.009; 0.8) | -0.57 (-1.0; -0.1) | 0.16 (-0.3; 0.6) |
| Cases were patients with cancer and VTE, whereas controls were patients with cancer without VTE. Abbreviation: CI: confidence interval.  *p<0.05; ** p<0.001 | | | | | | | | | | |

**Supplementary Table 7. Differences in the EQ-5D-3L results in patients with venous thromboembolic disease according to tumor site**

| **EQ-5D-3L** |  | **Index score** |
| --- | --- | --- |
| Gynecologic | Cases | 0.23 |
|  | Controls | 0.72 |
|  | Mean difference | -0.48** |
|  | Effect size (95% CI) | 0.49 (0.2; 3.0) |
|  | Relative efficiency (95% CI) | -1.69 (2.7; -0.7) |
| Lung | Cases | 0.69 |
|  | Controls | 0.78 |
|  | Mean difference | -0.09 |
|  | Effect size (95% CI) | 1.31 (0.6; 2.9) |
|  | Relative efficiency (95% CI) | -0.35 (-0.9; 0.2) |
| Digestive | Cases | 0.60 |
|  | Controls | 0.78 |
|  | Mean difference | -0.18** |
|  | Effect size (95% CI) | 2.53* (1.3; 5.2) |
|  | Relative efficiency (95% CI) | -0.65 (-1.1; -0.1) |
| Urological | Cases | 0.50 |
|  | Controls | 0.84 |
|  | Mean difference | -0.34* |
|  | Effect size (95% CI) | 7.4* (1.6; 65.6) |
|  | Relative efficiency (95% CI) | -1.28 (-2.5, -0.03) |
| Lymphoma | Cases | 0.57 |
|  | Controls | 0.77 |
|  | Mean difference | -0.20* |
|  | Effect size (95% CI) | 1.1 (0.52; 2.4) |
|  | Relative efficiency (95% CI) | -0.65 (-1.2; 0.05) |
| Other sites | Cases | 0.46 |
|  | Controls | 0.77 |
|  | Mean difference | -0.31** |
|  | Effect size (95% CI) | 2.55* (1.4; 4.9) |
|  | Relative efficiency (95% CI) | -0.85 (-1.3; -0.4) |
| Cases were patients with cancer and VTE, whereas controls were patients with cancer without VTE. Abbreviation: CI: confidence interval. *p<0.05; ** p<0.001 | | |

**Supplementary Table 8. Differences in the VEINES-QOL/Sym results in patients with venous thromboembolic disease according to tumor site**

| **VEINES-QOL/Sym** |  | **VEINES-QoL** | **VEINES/Sym** |
| --- | --- | --- | --- |
| Gynecologic | Controls | 49.6 | 48.4 |
|  | Cases | 43.7 | 43.2 |
|  | Mean difference | -5.8 | -5.2 |
|  | Effect size (95% CI) | 0.81 (0.06; 3.0) | 0.46 (0.03; 1.7) |
|  | Relative efficiency (95% CI) | 0.51 (-0.6; 1.6) | 0.40 (-0.7; 1.5) |
| Lung | Controls | 53.3 | 52.8 |
|  | Cases | 45.7 | 47.9 |
|  | Mean difference | -7.6** | -4.9* |
|  | Effect size (95% CI) | 1.17 (0.4; 2.5) | 1.40 (0.5; 3.0) |
|  | Relative efficiency (95% CI) | 1.06 (0.4; 1.7) | 0.66 (0.06; 1.3) |
| Digestive | Controls | 50.4 | 50.4 |
|  | Cases | 38.2 | 40.4 |
|  | Mean difference | -12.2** | -10.0** |
|  | Effect size (95% CI) | 0.87 (0.3; 1.8) | 1.14 (0.4; 2.2) |
|  | Relative efficiency (95% CI) | 1.4 (0.8; 2.0) | 0.99 (0.4; 1.5) |
| Urological | Controls | 48.5 | 49.2 |
|  | Cases | 45.5 | 46.6 |
|  | Mean difference | -3.0 | -2.6 |
|  | Effect size (95% CI) | 0.74 (0.0007; 4.6) | 0.36 (0.0003; 2.2) |
|  | Relative efficiency (95% CI) | 0.35 (-1.2; 2.0) | 0.27 (-1.3; 1.88) |
| Lymphomas | Controls | 53.1 | 52.0 |
|  | Cases | 42.3 | 42.5 |
|  | Mean difference | -10.7** | -9.5** |
|  | Effect size (95% CI) | 0.41 (0.12; 0.97) | 0.35 (0.1; 0.8) |
|  | Relative efficiency (95% CI) | 1.39 (0.7; 2.1) | 0.99 (0.28; 1.7) |
| Other sites | Controls | 52.4 | 52.5 |
|  | Cases | 44.6 | 44.0 |
|  | Mean difference | -7.8** | -8.5* |
|  | Effect size (95% CI) | 0.92 (0.3; 2.0) | 1.07 (0.4; 2.3) |
|  | Relative efficiency (95% CI) | 1.12 (0.5; 1.7) | 0.96 (0.3; 1.7) |
| Cases were patients with cancer and VTE, whereas controls were patients with cancer without VTE. Abbreviation: CI: confidence interval. *p<0.05; ** p<0.001 | | | |

**Supplementary Table 9. Differences in the PEmb-QoL score results in patients with venous thromboembolic disease according to tumor site**

| **PEmb-QoL** |  | **Quality of life** | **Frequency of complaints** | **Limitations in daily life activities** | **Work-related problems** | **Social limitations** | **Intensity of complaints** | **Emotional complaints** |
| --- | --- | --- | --- | --- | --- | --- | --- | --- |
| Gynecologic | Cases | 67.9 | 59.4 | 96.3 | 100 | 50.0 | 40.0 | 62.0 |
|  | Controls | 25.6 | 29.6 | 29.5 | 38.9 | 17.0 | 19.0 | 19.4 |
|  | Mean difference | +42.3 | +27.8 | +66.8* | +61.1 | +33.0 | +21.0 | +42.6 |
|  | Effect size (95% CI) | - | - | - | - | - | - | - |
|  | Relative efficiency (95% CI) | - | - | - | - | - | - | - |
| Lung | Cases | 35.6 | 28.6 | 37.3 | 61.5 | 32.7 | 27.7 | 25.7 |
|  | Controls | 21.6 | 20.9 | 26.3 | 36.8 | 14.3 | 14.6 | 17.7 |
|  | Mean difference | +14.0 | +7.7 | +11.0 | +24.7 | +18.3* | +13.1 | +8.0 |
|  | Effect size (95% CI) | 0.98 (0.4; 2.8) | 1.3 (0.6; 3.8) | 0.43  (0.2; 1.2) | 0.97 (0.4; 2.8) | 1.3 (0.6; 3.8) | 1.05 (0.5; 3.0) | 0.88 (0.4; 2.5) |
|  | Relative efficiency (95% CI) | 0.60 (-0.02; 1.2) | 0.32 (-0.3; 0.9) | 0.39 (-0.2; 1.0) | 0.55 (-0.07; 1.2) | 0.7 (0.06; 1.3) | 0.59 (-0.02; 1.2) | 0.36 (-0.2; 0.9) |
| Digestive | Cases | 40.2 | 34.4 | 45.8 | 71.9 | 25.0 | 27.5 | 36.5 |
|  | Controls | 22.8 | 19.8 | 30.8 | 40.5 | 12.8 | 16.2 | 16.0 |
|  | Mean difference | +17.4* | +14.6 | +15.0 | +31.4 | +12.2 | +11.3 | +20.4* |
|  | Effect size (95% CI) | 0.38 (0.1; 1.6) | 0.53 (0.2; 2.2) | 0.63 (0.2; 2.6) | 1.00 (0.4; 4.2) | 1.42 (0.6; 6.0) | 0.60 (0.2; 2.5) | 0.7 (0.3; 2.9) |
|  | Relative efficiency (95% CI) | 0.80 (0.04; 1.5) | 0.63 (-0.1; 1.4) | 0.54 (-0.2; 1.3) | 0.7 (-0.05; 1.4) | 0.53 (-0.2; 1.3) | 0.56 (-0.2; 1.3) | 0.9 (0.2; 1.7) |
| Urological | Cases | 45.0 | 27.1 | 51.8 | 100 | 16.6 | 26.6 | 48.0 |
|  | Controls | 24.6 | 22.5 | 26.4 | 36.5 | 15.4 | 16.9 | 17.8 |
|  | Mean difference | +20.4 | +4.5 | +25.4 | +63.5* | +1.2 | +9.7 | +30.2 |
|  | Effect size (95% CI) | 0.15 (0.03; 5.9) | 0.47 (0.1; 18,8) | 0.25 (0.05; 9.9) | 0.0* | 0.3 (0.06; 12.0) | 2.20 (0.4; 86) | 2.2 (0.4; 86) |
|  | Relative efficiency (95% CI) | 0.83 (-0.5; 2.2) | 0.17 (-1.1; 1.5) | 1.00 (-0.3; 2.4) | 1.5  (0.06;3.0) | 0.05  (-1.3; 1.4) | 0.43 (-0.9; 1.8) | 1.20 (-0.2; 2.6) |
| Lymphoma | Cases | 43.1 | 31.9 | 49.8 | 66.7 | 33.3 | 38.9 | 38.2 |
|  | Controls | 24.1 | 22.7 | 32.0 | 39.1 | 20.0 | 15.2 | 15.3 |
|  | Mean difference | +19.0* | +9.2 | +17.8 | +27.6 | +13.3 | +23.7* | +22.9* |
|  | Effect size (95% CI) | 1.9 (0.7; 7.4) | 1.29 (0.5; 4.9) | 1.91 (0.7; 7.3) | 1.2 (0.5; 4.5) | 1.2 (0.5; 4.6) | 2.3 (0.9; 8.8) | 2.5* (1.0; 9.6) |
|  | Relative efficiency (95% CI) | 0.79 (0.04; 1.5) | 0.32 (-0.4; 1.0) | 0.6 (-0.1; 1.3) | 0.59 (-0.1; 1.3) | 0.4 (-0.3; 1.1) | 0.95 (0.2; 1.7) | 1.1 (0.4; 1.9) |
| Other sites | Cases | 53.3 | 38.3 | 66.9 | 80.0 | 53.3 | 40.7 | 40.5 |
|  | Controls | 22.2 | 17.0 | 28.8 | 42.0 | 15.7 | 13.7 | 17.2 |
|  | Mean difference | +31.1** | +21.3** | +38.1** | +38.0** | +37.6** | +27.0** | +23.3** |
|  | Effect size (95% CI) | 0.91 (0.4; 2.4) | 0.71 (0.3; 1.8) | 1.09 (0.5; 2.8) | 0.66 (0.3; 1.7) | 1.63 (0.8; 4.2) | 1.44 (0.7; 3.7) | 1.68 (0.8; 4.4) |
|  | Relative efficiency (95% CI) | 1.4 (0.8; 2.0) | 0.9 (0.3; 1.5) | 1.3 (0.7; 1.9) | 0.8 (0.2; 1.4) | 1.25 (0.6; 1.8) | 1.43 (0.8; 2.0) | 1.1 (0.5; 1.7) |
| Cases were patients with cancer and VTE, whereas controls were patients with cancer without VTE. Abbreviation: CI: confidence interval.  *p<0.05; ** p<0.001 | | | | | | | | |
